# MCIndoor20000: A fully-labeled image dataset to advance indoor objects detection

**DOI:** 10.1016/j.dib.2017.12.047

**Published:** 2018-01-03

**Authors:** Fereshteh S. Bashiri, Eric LaRose, Peggy Peissig, Ahmad P. Tafti

**Affiliations:** a Department of Electrical Engineering, University of Wisconsin-Milwaukee, WI, USA; b Biomedical Informatics Research Center, Marshfield Clinic Research Institute, WI, USA

**Keywords:** Image dataset, Large-scale dataset, Image classification, Supervised learning, Indoor objects, Deep learning

## Abstract

A fully-labeled image dataset provides a unique resource for reproducible research inquiries and data analyses in several computational fields, such as computer vision, machine learning and deep learning machine intelligence. With the present contribution, a large-scale fully-labeled image dataset is provided, and made publicly and freely available to the research community. The current dataset entitled *MCIndoor20000* includes more than 20,000 digital images from three different indoor object categories, including doors, stairs, and hospital signs. To make a comprehensive dataset addressing current challenges that exist in indoor objects modeling, we cover a multiple set of variations in images, such as rotation, intra-class variation plus various noise models. The current dataset is freely and publicly available at https://github.com/bircatmcri/MCIndoor20000.

**Specifications table**

**Table:** 

Subject area	Machine learning, computer vision, deep learning, machine intelligence.
More specific subject area	Object classification, object detection, object recognition.
Type of data	2D-RGB digital images (.JPEG, .PNG).
How data was acquired	Original images were collected in Marshfield Clinic by capturing photos from remarkable landmark objects, including clinic signs, doors and stairs. Images are manually cropped to eliminate the effect of surrounding objects in the learning process. To cover multiple variations in the objects model, we systematically rotated and augmented diverse noises to the original images.
Data format	Digital images, in raw and processed formats.
Data source location	Marshfield Clinic, Marshfield, Wisconsin, USA.
Data accessibility	The dataset is accessible at [1], and it is freely and publicly available for any academic, educational, and research purposes.

**Value of the data**

• Machine intelligence and particularly computational vision have become ubiquitous in our daily life, with a variety of applications ranging from face recognition and fingerspelling to surveillance systems and healthcare informatics. Core to many of these applications is image classification and recognition which is defined as an automatic task that assigns a label from a fixed set of categories to an input image. The MCIndoor20000 dataset is a resource for use by the computer vision and deep learning community, and it advances image classification research.
• The MCIndoor20000 dataset, collected in Marshfield Clinic, Marshfield, presents various digital images of three guideline indoor objects, including clinic signs, doors and stairs.
• To provide a comprehensive image classification repository, the current dataset covers several object model variations involved from the perspectives of computer vision and deep learning strategies. The variations include viewpoint variation, intra-class variation, rotation, noisy conditions (e.g., Gaussian, Poisson), and occlusion.
• The present dataset assists reproducible research and allows rapid application development (RAD) and fast prototyping by the research community.

## 1 Data

**Fig. 1:**
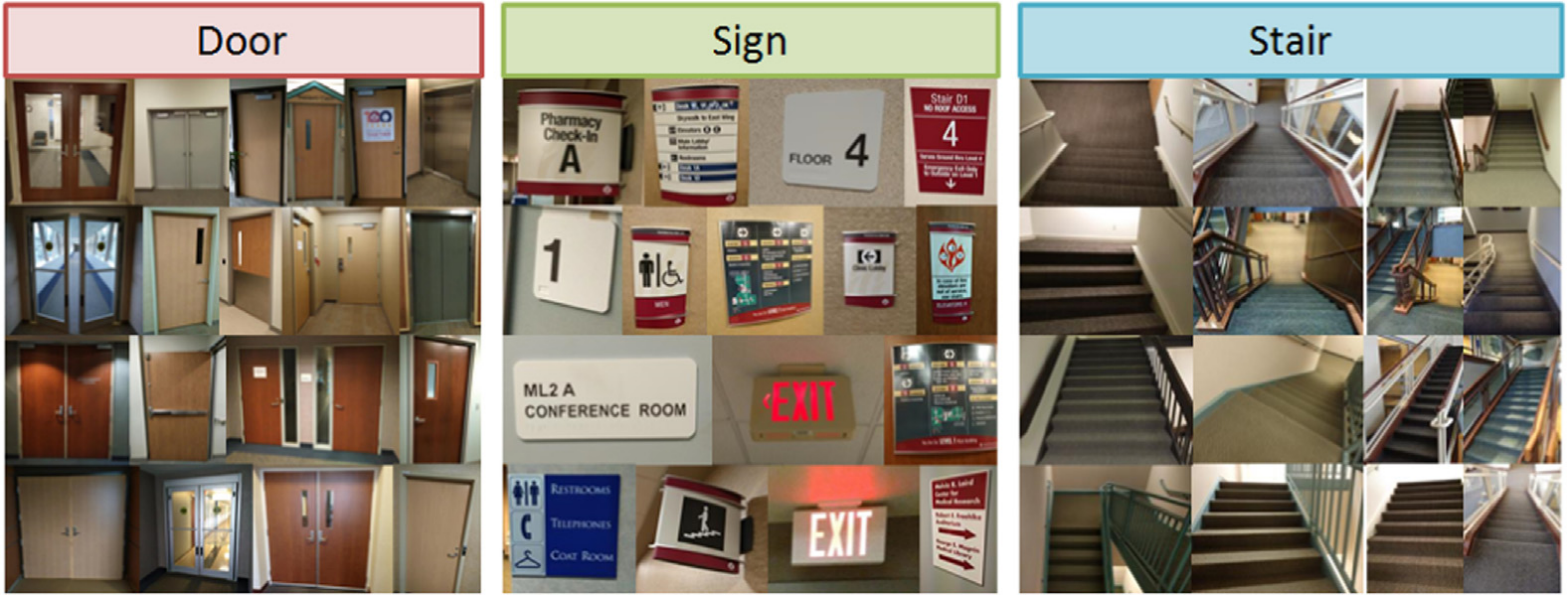
Sample images from the categories covered by the MCIndoor20000.

The MCIndoor20000 is a fully-labeled image dataset that was launched in Marshfield Clinic to facilitate broad use of image classification and recognition. Examples of such valuable annotated image datasets include OpenImages [2], CIFAR-10 and CIFAR-100 [3], [4], ImageNet [5] as well as environmental scene database [6]. The uniqueness of the MCIndoor20000 is that the dataset consists of three different image categories, including: (1) Door, (2) Sign, and (3) Stair, all of which are remarkable landmarks for indoor navigation. The number of original images is 754, 702 and 599 across these categories, respectively. Fig. 1 presents different examples from each category. One potential application of the present dataset lies in the development of combined machine learning and computer vision algorithms to help people with visual impairment during mobility, especially in unfamiliar environments, such as hospitals and urgent cares [7].

**Fig. 2:**
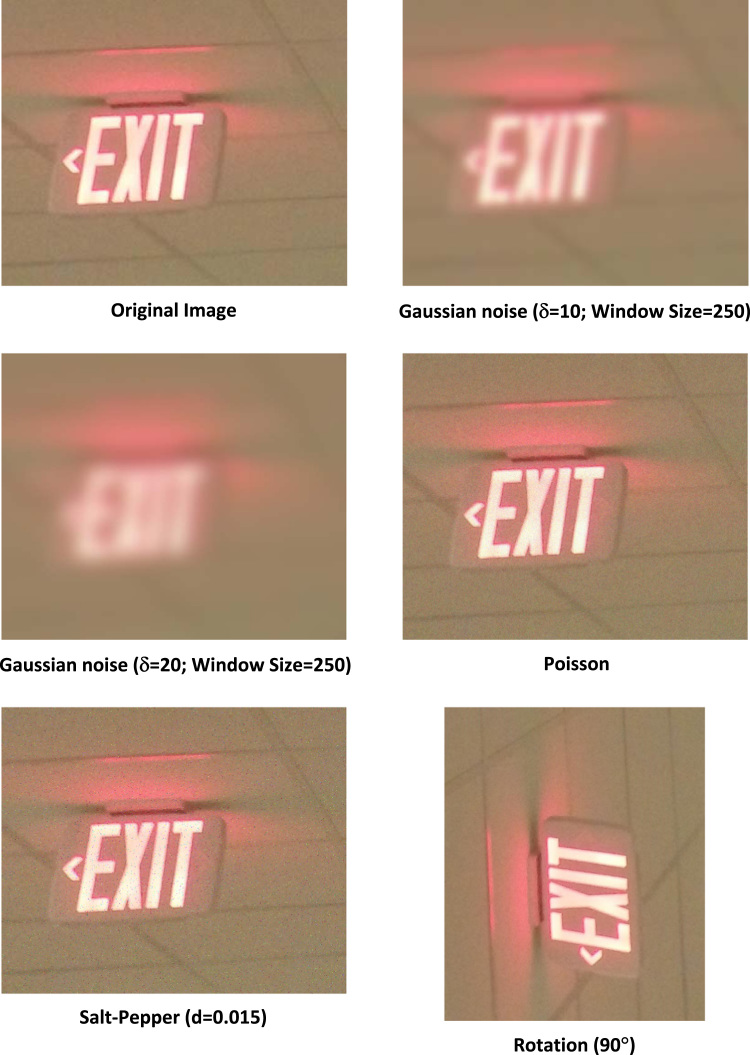
The MCIndoor20000 dataset carries several objects model variations.

The dataset's original images were captured in Marshfield Clinic in summer 2017, with a variety of viewpoint and intra-class variations along with occlusion across each class. We then systematically added Gaussian, Poisson, and Salt-Pepper noises to the original images, rotating all images to make a comprehensive dataset. Fig. 2 shows an example of all the variations that exist in the dataset. With respect to the Gaussian noise model, a rotationally symmetric Gaussian lowpass filter of size 250 with standard deviation *δ* of 10 and 20 has been applied to the original images. The salt-pepper noise model was configured by the noise density of 0.015. The Poisson noise model usually generates Poisson noise with the use of data. As the MCIndoor20000 input images are all Unit8, therefore the input pixel values are used directly without scaling.

To aid in the reproducibility of image classification and object recognition we have collected, labeled, generated, and published the images in the MCIndoor20000 dataset [1]. Based on the results received when training AlexNet using MCIndoor20000, the dataset provides high quality and diverse images that are shown to be sufficient to train the classification algorithms to identify doors, signs and stairs from an indoor setting. This dataset can be used as is or combined with other image datasets in order to provide more robust classification of indoor objects.

MCIndoor20000 does have some limitations. Although the dataset has a variety of doors, signs and staircases, the images were all collected from a single organization's facility, so variations in style within each subclass may be somewhat limited. There is also a limitation with classes that are similar but are not represented in the dataset. Such similarities would include things such as escalators and stairs or posters and signs. The dataset includes the variations introduced by different types of noise and rotations, but does not include variations in illumination or deformation that might be caused by the environment or the capture device. We believe these limitations are minor and could be addressed in future datasets or by augmenting the existing dataset with other such datasets.

## 2 Materials, methods and experimental validations

To analyze the quality and quantity attributes of the MCIndoor20000 dataset, we utilized a widely-used pre-trained deep convolutional neural network (CNN) model, namely AlexNet [8]. The AlexNet model is trained with 1.2 million high resolution images from 1000 different classes and has shown promising results in image recognition [8]. In this experiment, the knowledge that is obtained from learning a large dataset is used for classification of images of interest. In the machine learning community, this task in which a set of learned features of a network is transferred to a new problem is called Transfer Learning [9].

A small portion of the MCIndoor20000 dataset is selected randomly and is used as a training set. The last few layers of the pre-trained CNN model are fine-tuned for the new classification problem and a 3-way softmax layer and a classification output layer are added. Then, the annotated labels of the training images are employed to retrain the model. For the purpose of evaluating the performance of the trained network for image recognition the rest of the collected dataset is used. The experiment is repeated with different percentages of the total dataset used for the purpose of training as the results are presented in Table 1. Results illustrated in Table 1 show higher accuracy with the use of MCIndoor20000 rather than original images only, and it is statistically significant at *P*< 0.05.

**Table 1:** An experimental validation performed on the dataset. The MCIndoor20000 dataset includes the original images along with all variations discussed in the Data section. The MCIndoor2000 (Original Images) includes only the original images without any variations. “ACC” stands for accuracy, while “TPR” and “PPV” stands for true positive rate and positive predictive value, respectively.

**Dataset ID**	**Train/Test Percentage**	**ACC**	**TPR**	**PPV**
MCIndoor20000	20% Train, 80% Test	99.8%	99.8%	99.8%
MCIndoor20000	10% Train, 90% Test	99.6%	99.6%	99.6%
MCIndoor2000 (Original Images)	20% Train, 80% Test	90.4%	89.8%	92.7%
MCIndoor2000 (Original Images)	10% Train, 90% Test	64.4%	63.3%	75.0%
