# Supplementary material for: MCIndoor20000: A fully-labeled image dataset to advance indoor objects detection
Source: Data Brief. 2018 Jan 3;17:71–5. doi: 10.1016/j.dib.2017.12.047 (PMC5988436; doi:10.1016/j.dib.2017.12.047)
Supplement: Supplementary file 1 — Supplementary material [file mmc1.docx]

**Declarations of interest**

none.
